# Effectiveness and safety of telitacicept in refractory ocular myasthenia gravis: a retrospective, real-world study

**DOI:** 10.3389/fimmu.2026.1797461

**Published:** 2026-09-03

**Authors:** Chao Sun, Danni Liu, Yonglan Tang, Biying Zhang, Rongjing Guo, Ting Gao, Xiangqi Cao, Sijia Hao, Qingqing Wang, Hui Yu, Ting Chang

**Affiliations:** Department of Neurology, Tangdu Hospital, The Fourth Military Medical University, Xi’an, China

**Keywords:** refractory ocular myasthenia gravis, biologic therapy, telitacicept, effectiveness, safety

## Abstract

**Introduction:**

Refractory ocular myasthenia gravis (OMG) remains a significant therapeutic challenge with limited evidence-based biologic therapies. This study aimed to evaluate the effectiveness and safety of telitacicept, a recombinant B-lymphocyte stimulator receptor-antibody fusion protein, in refractory OMG.

**Methods:**

This retrospective, single-center observational study assessed clinical outcomes, immunological biomarkers, and safety following add-on telitacicept therapy in refractory OMG. Disease severity was evaluated using the Myasthenia Gravis Activities of Daily Living (MG-ADL), Quantitative Myasthenia Gravis (QMG), and Myasthenia Gravis Impairment Index (MGII) scales. Serum immunoglobulins, complement components, and CD19^+^ B-cell counts were measured, and adverse events were systematically recorded.

**Results:**

Twelve patients with refractory OMG were included. Median baseline MG-ADL, QMG, and MGII scores were 5.5 (interquartile range [IQR] 5.0–6.0), 7.0 (IQR 5.3–8.0), and 16.0 (IQR 12.0–18.0), respectively. Statistically significant improvement was observed as early as week 1 (all *p* < 0.05). By week 12, MG-ADL scores decreased by a median of 3.0 (95% CI: 2.0–4.0; *p* < 0.001, r = 0.89), with consistent improvements in QMG and MGII scores (all *p* < 0.01, all r > 0.85). Minimal symptom expression (MSE) was achieved in 7 of 12 patients (58.3%) within the 12-week period. The median daily prednisone dose was reduced from 15.0 mg (IQR 10.0–22.5) to 9.0 mg (IQR 5.0–13.8) by week 12 (median decrease: 7.5 mg, 95% CI: 2.5–13.5; *p* = 0.031, r = 0.79), with 1 patient discontinuing corticosteroids by week 8. Telitacicept treatment was associated with reductions in serum IgG, IgA, and IgM levels, alongside increased levels of complement components. No serious adverse events, infections, or treatment discontinuations occurred.

**Conclusion:**

Telitacicept was associated with rapid, sustained clinical improvement and a favorable safety profile in refractory OMG, which supports its potential role as an add-on therapeutic option.

## Introduction

Myasthenia gravis (MG) is an acquired autoimmune disorder of the neuromuscular junction characterized by fluctuating, fatigable skeletal muscle weakness mediated by pathogenic autoantibodies, complement activation, and T-cell-mediated immune responses (1, 2). Ocular myasthenia gravis (OMG) represents a distinct clinical subtype in which weakness is confined to the extraocular muscles, levator palpebrae, and orbicularis oculi, manifesting predominantly as ptosis and diplopia (3). Although OMG is not life-threatening, persistent ocular symptoms impose a substantial burden on patients, leading to impaired visual function, reduced work capacity, and marked deterioration in quality of life (4, 5). Importantly, OMG is not a benign condition; while approximately 20-30% of patients remain restricted to ocular involvement, a significant proportion progress to generalized myasthenia gravis (GMG) over time, underscoring the clinical relevance of early and effective therapeutic intervention (6).

Despite evidence demonstrating that early initiation of corticosteroids and immunosuppressive therapy can improve ocular symptoms and may reduce the risk of generalization, treatment outcomes in OMG remain suboptimal (7, 8). Approximately 60% of patients fail to achieve pharmacological remission or minimal manifestations, and many experience treatment-related adverse events (TRAEs) with long-term corticosteroid or immunosuppressant use (9). By extrapolation from refractory GMG, refractory OMG can be defined by inadequate response to corticosteroids and conventional immunosuppressive agents, relapse or worsening of ocular symptoms during steroid tapering, or intolerance to TRAEs (6). Although refractory OMG is not typically life-threatening, persistent diplopia and ptosis substantially impair daily functioning, work performance, and the quality of life, remaining a real-world therapeutic challenge.

Advances in the understanding of MG pathogenesis have led to the development of biologic therapies that have transformed the management of refractory generalized disease. Neonatal Fc receptor antagonists, e.g., efgartigimod (10), and complement C5 inhibitors, e.g., eculizumab (11), are currently approved for GMG; however, evidence supporting their efficacy in isolated ocular disease remains limited to subgroup analyses and case reports, with no therapy specifically approved for refractory OMG. Moreover, existing biologic agents address only selected pathogenic mechanisms and may be ineffective or poorly tolerated in a subset of patients. Given the central role of autoreactive B cells in MG, through antibody production, antigen presentation, and cytokine secretion (12), current B-cell-directed approaches also have limitations. Anti-CD20 therapy with rituximab does not eliminate long-lived plasma cells and is associated with an increased risk of serious infections, particularly in individuals with long disease course (13, 14), while CD19-directed chimeric antigen receptor T-cell therapy, despite its mechanistic appeal, remains constrained by high cost and limited accessibility (15). Collectively, these observations underscore the ongoing need for novel mechanism-based therapeutic approaches for clinical refractory OMG.

Telitacicept is a recombinant fusion protein comprising the extracellular domain of transmembrane activator and calcium modulator and cyclophilin ligand interactor (TACI) fused to the Fc portion of human IgG1, enabling simultaneous neutralization of B-lymphocyte stimulator (BLyS/BAFF) and a proliferation-inducing ligand (APRIL) (16). By targeting these two key cytokines that govern B-cell survival, differentiation, and plasma cell maintenance, telitacicept exerts upstream immunomodulatory effects, suppressing the generation of autoreactive memory B cells and antibody-secreting plasma cells and thereby reducing pathogenic autoantibody production (16). In GMG, telitacicept has demonstrated robust clinical efficacy and a favorable safety profile, with phase II data showing significant reductions in Quantitative Myasthenia Gravis (QMG) scores and corticosteroid requirements (17). Furthermore, supported by phase III study data (NCT05737160), telitacicept was approved by the National Medical Products Administration (NMPA) in May 2025 for the treatment of adults with anti-acetylcholine receptor (AChR) antibody-positive GMG. Post-hoc analyses have suggested potential benefits for the ocular symptoms. Recently, Lin et al. reported a real-world case series demonstrating the effectiveness of add-on telitacicept in seven patients with refractory ocular symptoms. Although this study provides valuable preliminary evidence, it is limited by a small sample size, population heterogeneity (including both OMG and GMG), non-standard dosing (e.g., 80 mg outside the phase II criteria), and a lack of early dynamic monitoring (18). We therefore conducted this single-center, retrospective study to summarize our preliminary, real-world experience regarding the effectiveness and safety of telitacicept as an add-on therapy in 12 refractory OMG patients, with dynamic efficacy evaluations starting from the first week of treatment.

## Methods

### Participants and study design

This retrospective, single-center observational study was conducted at the Department of Neurology, Tangdu Hospital, Fourth Military Medical University, between June and December 2025. The diagnosis of MG was established based on the presence of fluctuating, fatigable muscle weakness in combination with at least one of the following criteria: seropositivity for anti-AChR antibodies, a positive neostigmine test, abnormal electrophysiological findings on repetitive nerve stimulation and/or single-fiber electromyography.

Eligible participants were required to meet the following inclusion criteria: (1) a confirmed diagnosis of OMG for a minimum duration of three months, classified as Myasthenia Gravis Foundation of America (MGFA) class I; (2) an MGII PRO ocular subscore ≥6 at screening and baseline, with at least two ocular items scoring ≥2; (3) inadequate clinical response to at least two immunosuppressive therapies, intolerance to TRAEs, or relapse during corticosteroid tapering or withdrawal; and (4) administration of telitacicept as add-on therapy during the study period. Exclusion criteria comprised: (1) evidence of active or chronic infection, including human immunodeficiency virus infection, latent hepatitis B, or tuberculosis; (2) a prior diagnosis of GMG; (3) other potential causes of ptosis, diplopia, or peripheral muscle weakness (e.g., Graves’ ophthalmopathy, blepharospasm, progressive external ophthalmoplegia, muscular dystrophy, brainstem or cranial nerve lesions). All consecutive patients who met these criteria and received add-on telitacicept during the study period were included. The sample size was therefore determined by clinical availability, and no *a priori* power calculation was performed.

The study protocol was reviewed and approved by the Ethics Committee of Tangdu Hospital, Fourth Military Medical University (approval No. 202509-21). The studies were conducted in accordance with the local legislation and institutional requirements. Written informed consent was obtained from the individual(s) for the publication of any potentially identifiable images or data included in this article.

### Treatment, follow-up, and assessments

Patients were administered telitacicept subcutaneously at an initial dose of 160 mg or 240 mg once weekly, with each treatment cycle comprising four consecutive weekly injections. The starting dose was individualized according to disease severity and patient-specific considerations, including economic factors. Concomitant corticosteroids and other immunosuppressive agents were tapered in a stepwise manner based on clinical response and tolerability.

Patients were followed up regularly after treatment initiation, with evaluations performed weekly during the first month and monthly thereafter, either through in-person outpatient visits or remote follow-up via WeChat. Effectiveness assessments were conducted at baseline, defined as the last clinical evaluation prior to the first telitacicept administration, and at each subsequent scheduled follow-up. Safety assessments were initiated after the first telitacicept injection and continued throughout the treatment and follow-up periods. Effectiveness outcomes included the MG-ADL score, the QMG score, and the Myasthenia Gravis Impairment Index (MGII), all of which were systematically recorded at each assessment time point.

Clinical response was classified according to the Myasthenia Gravis Foundation of America Post-Intervention Status (MGFA-PIS) criteria into four categories: worse, defined as clinical deterioration following treatment; unchanged, defined as no meaningful clinical improvement; improved, defined as symptomatic improvement with residual manifestations; and minimal symptom expression (MSE), defined as an MG-ADL score of 0 or 1. Concomitant corticosteroids and immunosuppressive agents were tapered once a patient demonstrated an MG-ADL improvement of ≥1 point; most tapers were initiated after MSE was achieved. Corticosteroids were tapered before steroid-sparing immunosuppressive agents to minimize long-term steroid exposure while maintaining disease control. Safety was evaluated by monitoring the incidence, severity, and duration of adverse events (AEs) following telitacicept administration. All AEs were documented in accordance with the Common Terminology Criteria for Adverse Events, version 5.0.

### Endpoints

The primary effectiveness endpoint was the change in MG-ADL score from baseline to week 12. Secondary endpoints included: (1) changes in QMG and MGII scores from baseline to week 12; (2) changes in MG-ADL, QMG, and MGII scores from baseline to week 4; and (3) the proportion of patients achieving MSE (MGFA-PIS) at weeks 4 and 12. The safety endpoint was the incidence, severity, and type of adverse events, graded according to CTCAE v5.0.

### Blood sample collection and laboratory assessments

Peripheral venous blood samples were obtained at baseline, defined as prior to the first subcutaneous administration of telitacicept, and subsequently at 4-week intervals corresponding to scheduled monthly follow-up visits throughout the treatment period. Laboratory assessments comprised both flow cytometry and serological analyses to characterize peripheral B-cell subsets and humoral immune parameters.

CD19^+^ B-cell counts were quantified by flow cytometry using APC-conjugated anti-CD19 monoclonal antibodies (HIB19, Beijing Tongsheng Shidai Biotech). Serum immunoglobulin levels, including IgG, IgA, and IgM, as well as the complement components C3c, C4, and C1 inhibitor, were measured using commercial kits from Siemens Healthcare Diagnostics Products GmbH in accordance with the manufacturer’s instructions.

### Statistical analysis

All continuous variables were assessed for normality using the Shapiro–Wilk test. Continuous variables were described as median with interquartile ranges (IQRs). Categorical variables were presented as counts and percentages. Given the limited sample size, deviation from normality, and repeated-measures longitudinal design, non-parametric approaches were employed. Changes from baseline at each follow-up time point were assessed using the exact Wilcoxon signed-rank test. The Hodges–Lehmann estimator was used to estimate median paired differences with 95% confidence intervals (CIs). Effect sizes for Wilcoxon signed-rank tests were calculated as $r=Z/\sqrt{N}$.

Missing outcome data were handled by available case analysis without imputation; the proportion of missing data at each time point is summarized in **Supplementary Table 2**. As this was an exploratory study involving multiple time-point comparisons, no adjustment for multiplicity was performed. Accordingly, all P values are descriptive and should be interpreted with caution.

Graphical visualizations were generated to depict longitudinal trends and individual response patterns, including mean trajectories with 95% confidence intervals, box plots, individual and normalized trajectory plots, and heatmaps. All statistical tests were two-sided, and *p* < 0.05 was considered statistically significant. Statistical analysis was performed with the SPSS version 20.0 (IBM Corp., Armonk, NY, USA), and graphical visualizations were generated using Python Matplotlib version 3.9.4.

## Results

### Baseline characteristics of patients

Between June and December 2025, twelve consecutive patients with refractory OMG were enrolled in this retrospective study. The cohort included 7 men (58.3%) and 5 women, with a median age at disease onset of 43.5 years (IQR, 32.0–58.5) and a median disease duration of 66.0 months (IQR, 31.5–96.0). Eleven patients were positive for anti-AChR antibodies, while one was seronegative. Thymic abnormalities were identified in five patients, including thymoma (n=3), thymic cyst (n=1), and thymic hyperplasia (n=1); three of these patients had previously undergone thymectomy (**Table 1**).

**Table 1:** Baseline characteristics of patients with refractory ocular myasthenia gravis.

Patient	Sex	Age at onset, years	Disease duration, months	Autoantibody profile	Thymic pathology	Thymectomy	Residual ocular manifestations	Comorbidities	Telitacicept dose, mg/week
1	Female	61	12	Seronegative	Thymoma	No	Ptosis, diplopia	Thyroid disease	160
2	Male	38	19	AChR-Ab positive	None	No	Ptosis, diplopia	None	240
3	Male	48	276	AChR-Ab positive	None	No	Ptosis, diplopia	Hypertension, diabetes	240
4	Male	6	84	AChR-Ab positive	None	No	Ptosis, diplopia	None	160
5	Female	58	36	AChR-Ab positive	None	No	Ptosis, diplopia	Hypertension	240
6	Male	41	30	AChR-Ab + RyR-Ab positive	None	No	Ptosis, diplopia	None	240
7	Male	5	144	AChR-Ab positive	None	No	Ptosis, diplopia, limited ocular motility	None	160
8	Female	34	96	AChR-Ab + Titin-Ab + RyR-Ab positive	Thymic cyst	Yes	Ptosis, diplopia	None	240
9	Male	30	48	AChR-Ab positive	Thymic hyperplasia	No	Ptosis, diplopia	None	160
10	Female	59	36	AChR-Ab positive	None	No	Ptosis	None	240
11	Male	46	93	AChR-Ab positive	Thymoma	Yes	Ptosis, diplopia	None	240
12	Female	61	96	AChR-Ab positive	Thymoma	Yes	Ptosis	None	240

AChR-Ab, anti-acetylcholine receptor antibody; RyR-Ab, anti-ryanodine receptor antibody; Titin-Ab, anti-Titin antibody.

All patients had prior immunotherapy and met criteria for refractory disease. Conventional immunosuppressive regimens included azathioprine, tacrolimus and mycophenolate mofetil. Five patients had previous treatment with biologic therapies, including the neonatal Fc receptor inhibitor efgartigimod, the complement C5 inhibitor eculizumab, and the anti-CD20 monoclonal antibody rituximab. The reasons for telitacicept initiation included refractoriness to standard and/or biologic therapies, intolerance to immunosuppressant-related AEs, and substantial impairment in the quality of life (**Supplementary Table 1**).

At baseline, ten cases presented with both ptosis and diplopia, whereas two exhibited isolated ptosis. Telitacicept was administered as add-on therapy in all cases. One patient received a single treatment cycle after achieving MSE and discontinued therapy for economic reasons. The detailed number of evaluable patients at each time point, along with the corresponding proportions of missing data, is provided in **Supplementary Table 2**.

### Effectiveness of telitacicept

**Figure 1:**
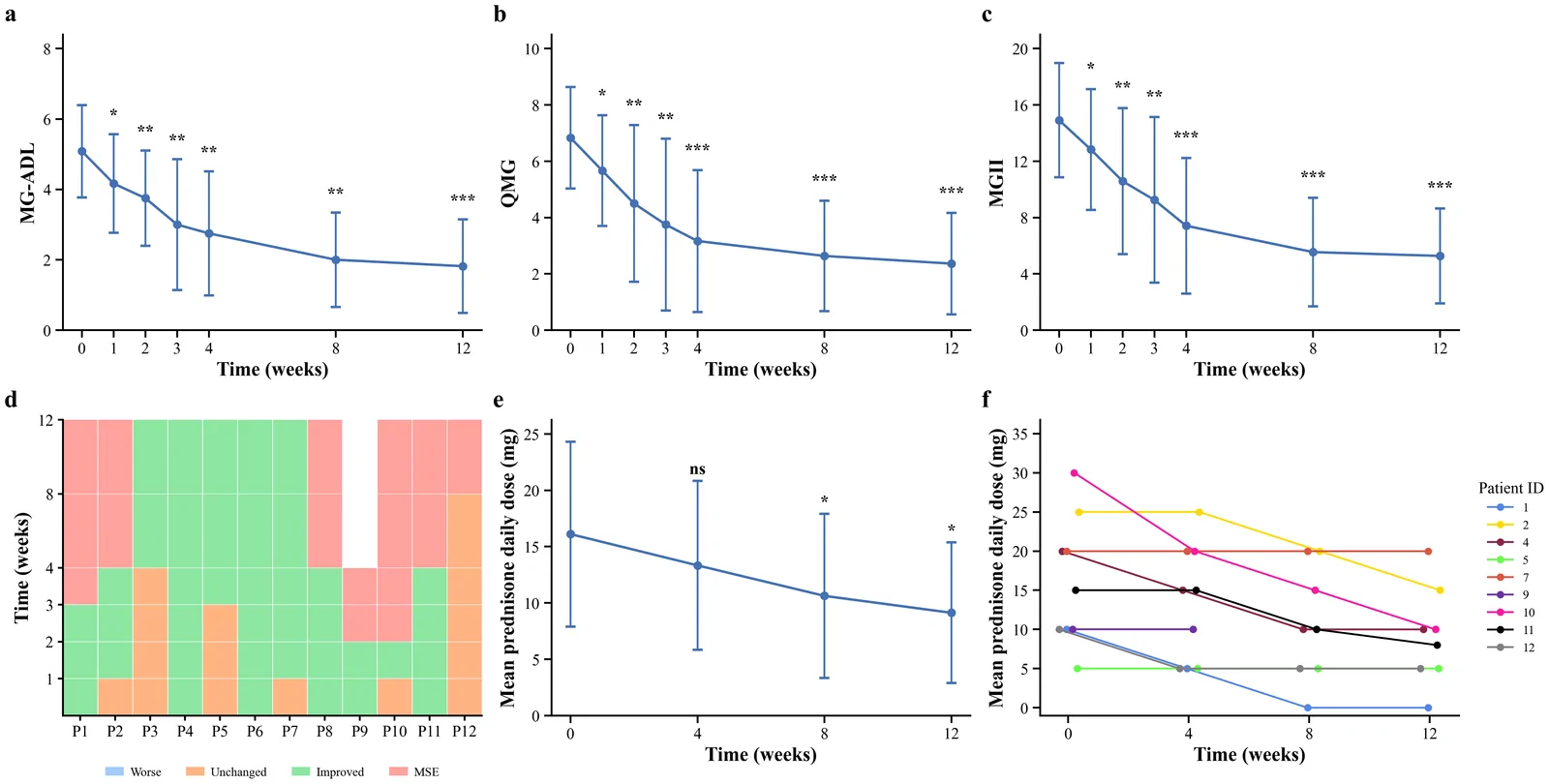
Clinical outcomes following telitacicept treatment in refractory ocular myasthenia gravis. **(a–c)** Changes from baseline in Myasthenia Gravis Activities of Daily Living (MG-ADL) **(a)**, Quantitative Myasthenia Gravis (QMG) **(b)**, and Myasthenia Gravis Impairment Index (MGII) **(c)** scores at weeks 1, 2, 3, 4, 8, and 12 after treatment initiation. **(d)** Proportion of the 12-week observation period during which each patient achieved minimal symptom expression (MSE). **(e, f)** Mean and individual daily prednisone doses from baseline through week 12. *P<0.05, **P<0.01, ***P<0.001.

At baseline, median MG-ADL, QMG, and MGII scores were 5.5 (interquartile range [IQR] 5.0–6.0), 7.0 (IQR 5.3–8.0), and 16.0 (IQR 12.0–18.0), respectively. Following telitacicept initiation, early reductions in ocular symptom severity scores were observed across the cohort (n = 12) at week 1. MG-ADL scores decreased to a median of 4.5 (within-subject median reduction = 0.8, 95% CI 0.0–2.0; exact *p* = 0.031, rank-biserial r = 0.64). Similarly significant early reductions were detected in QMG (median reduction = 1.0, 95% CI 0.0–2.0; *p* = 0.031, r = 0.64) and MGII (median reduction = 1.5, 95% CI 0.0–4.0; *p* = 0.016, r = 0.69) scores. These downward trends in clinical scale scores continued through week 4 (all *p* ≤ 0.002) (**Figure 1a**). At the 12-week follow-up (n=11), the reductions in clinical scores were sustained. The median MG-ADL score declined to 1.0 (IQR 1.0–3.0), reflecting a within-subject median reduction of 3.0 points (95% CI 2.0–4.0; *p* < 0.001, r = 0.89). Consistently, sustained reductions at week 12 were also recorded in QMG (median reduction = 4.5, 95% CI 3.5–5.5; *p* < 0.001, r = 0.89) and MGII (median reduction = 9.0, 95% CI 7.0–12.0; *p* < 0.001, r = 0.89) scores. Overall, MSE was achieved by 3 patients at week 4, an additional 3 at week 8, and 1 at week 12, totaling 7 patients (58.3%) during the 12-week follow-up period. By the end of the 12-week follow-up period, 6 of the 11 evaluated patients (54.5%) maintained MSE (**Figure 1d**). Concurrently, downward titrations in concomitant immunosuppressive therapies were recorded. The median daily prednisone dose was reduced from 15.0 mg (IQR 10.0–22.5) at baseline to 9.0 mg (IQR 5.0-13.8) by week 12 (median decrease: 7.5 mg, 95% CI: 2.5 to 13.5; *p* = 0.031, r = 0.79), with 1 patient completely discontinuing oral prednisone (**Figure 1e**). Among patients administered concomitant tacrolimus, the median daily dose decreased from 2.0 mg to 0.5 mg by week 12 (median reduction = 0.8 mg, 95% CI 0.3–1.5; *p* = 0.016, r = 0.79), with 3 patients discontinuing the drug entirely (**Figures 1e, f**).

**Figure 2:**
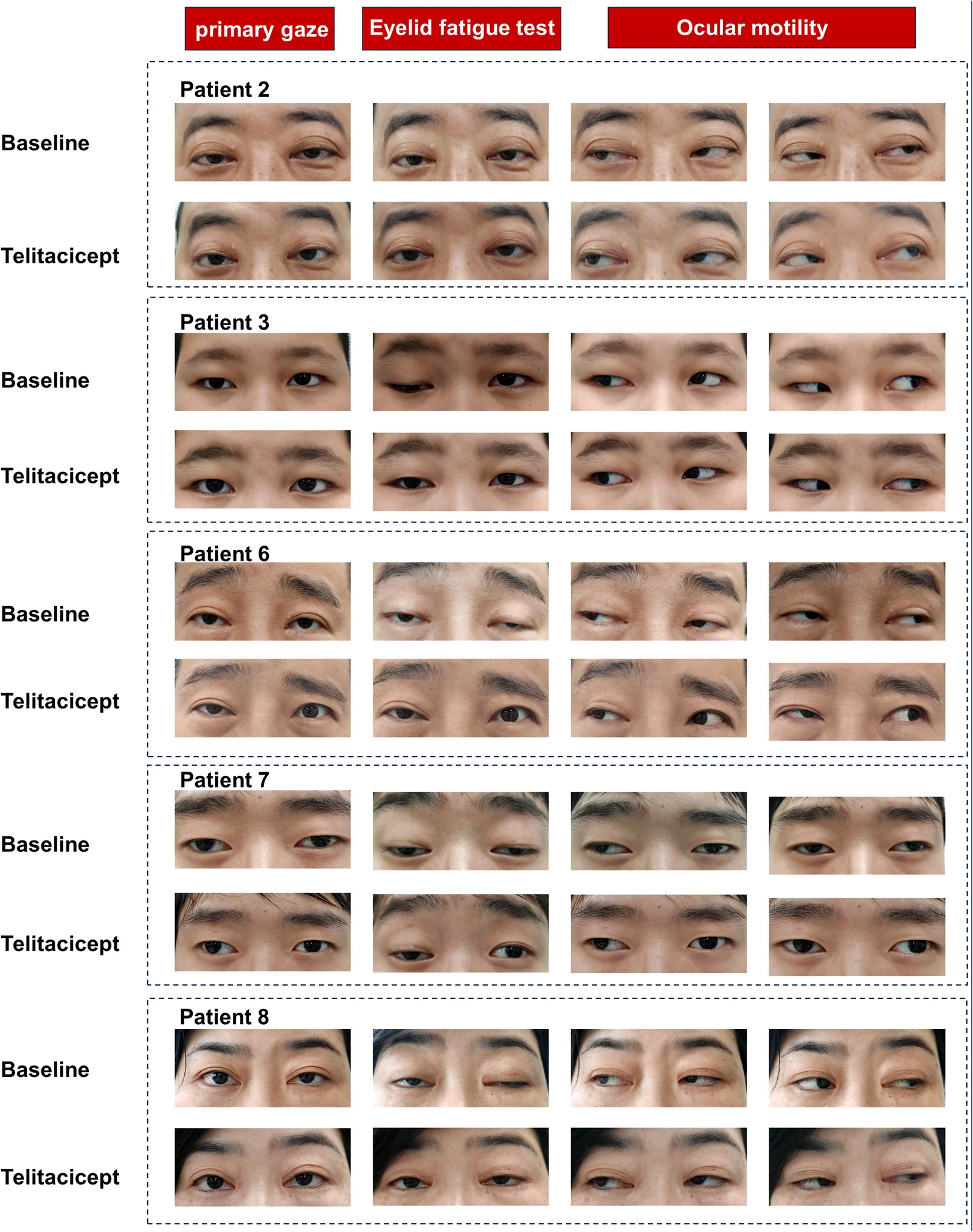
Representative clinical photographs depicting the course of ocular symptoms following telitacicept treatment. Photographs obtained before and after treatment illustrate changes in primary gaze (straight-ahead position), eyelid fatigability testing, and ocular motility.

Representative clinical photographs obtained at baseline and at the most recent follow-up visit demonstrated marked improvement in ptosis, as assessed by primary gaze, eyelid fatigability testing, and ocular motility examinations, further corroborating the reported clinical benefits of telitacicept therapy (**Figure 2**).

### Effects of telitacicept on ptosis and diplopia

**Figure 3:**
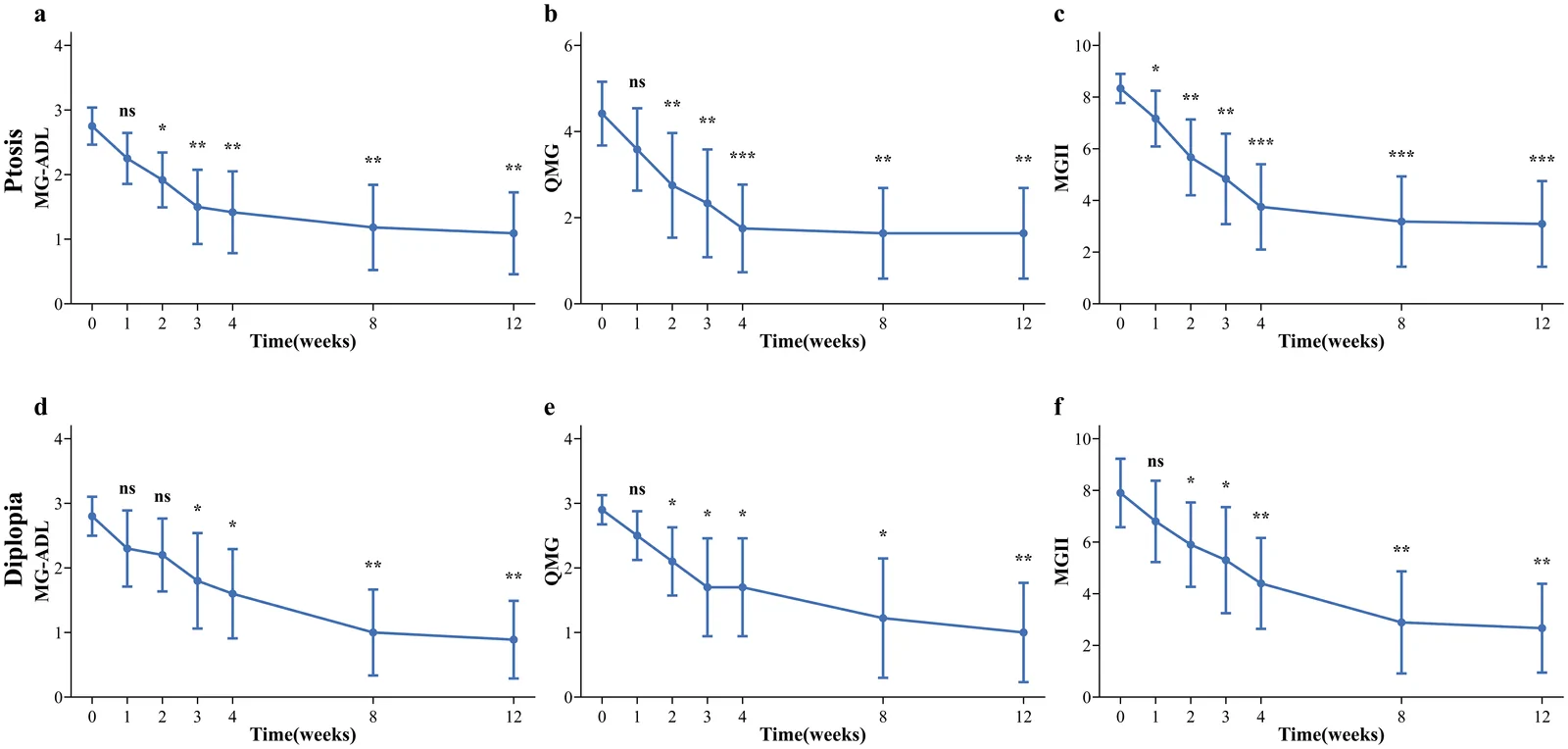
Differential effects of telitacicept on ptosis and diplopia in refractory ocular myasthenia gravis. **(a–c)** Changes from baseline in ptosis subscores in the Myasthenia Gravis Activities of Daily Living (MG-ADL), Quantitative Myasthenia Gravis (QMG), and Myasthenia Gravis Impairment Index (MGII) scales over the 12-week treatment period. **(d–f)** Changes from baseline in diplopia subscores in the MG-ADL, QMG, and MGII scales over the 12-week treatment period. *P<0.05, **P<0.01, ***P<0.001.

Ptosis and diplopia, the predominant clinical manifestations of refractory OMG, were longitudinally evaluated using the ocular subscores of the MG-ADL, QMG, and MGII scales. A downward trend in ptosis was detected as early as week 1, as reflected in the MGII ptosis subscore (median reduction = 1.0, 95% CI 0.0–2.0; *p* = 0.031, r = 0.64; **Figure 3c**). This reduction continued through week 4 (median reduction = 4.5, 95% CI 3.0–6.5; *p* = 0.001, r = 0.85; **Figure 3c**). In contrast, statistically significant reductions in diplopia subscores were not recorded until week 2. Similarly, based on the MG-ADL scale, ptosis subscores exhibited a reduction by week 2 (median reduction = 1.0, 95% CI 0.0–1.5; *p* = 0.016, r = 0.70; **Figure 3a**), with further declines by week 4 (median reduction = 1.5, 95% CI 0.5–2.0; *p* = 0.008, r = 0.74; **Figure 3a**). Notably, MG-ADL diplopia scores only showed a trend toward reduction at week 2 without reaching statistical significance (median reduction = 0.5, 95% CI 0.0–1.0; *p* = 0.063, r = 0.67; **Figure 3d**). Based on QMG scores, both ptosis and diplopia showed early downward trajectories at week 1, though these initial changes did not reach statistical significance (ptosis: median reduction = 1.0, 95% CI 0.0–1.5, *p* = 0.063, r = 0.60; diplopia: median reduction = 0.5, 95% CI 0.0–1.0, *p* = 0.125, r = 0.63, **Figure 3**).

**Figure 4:**
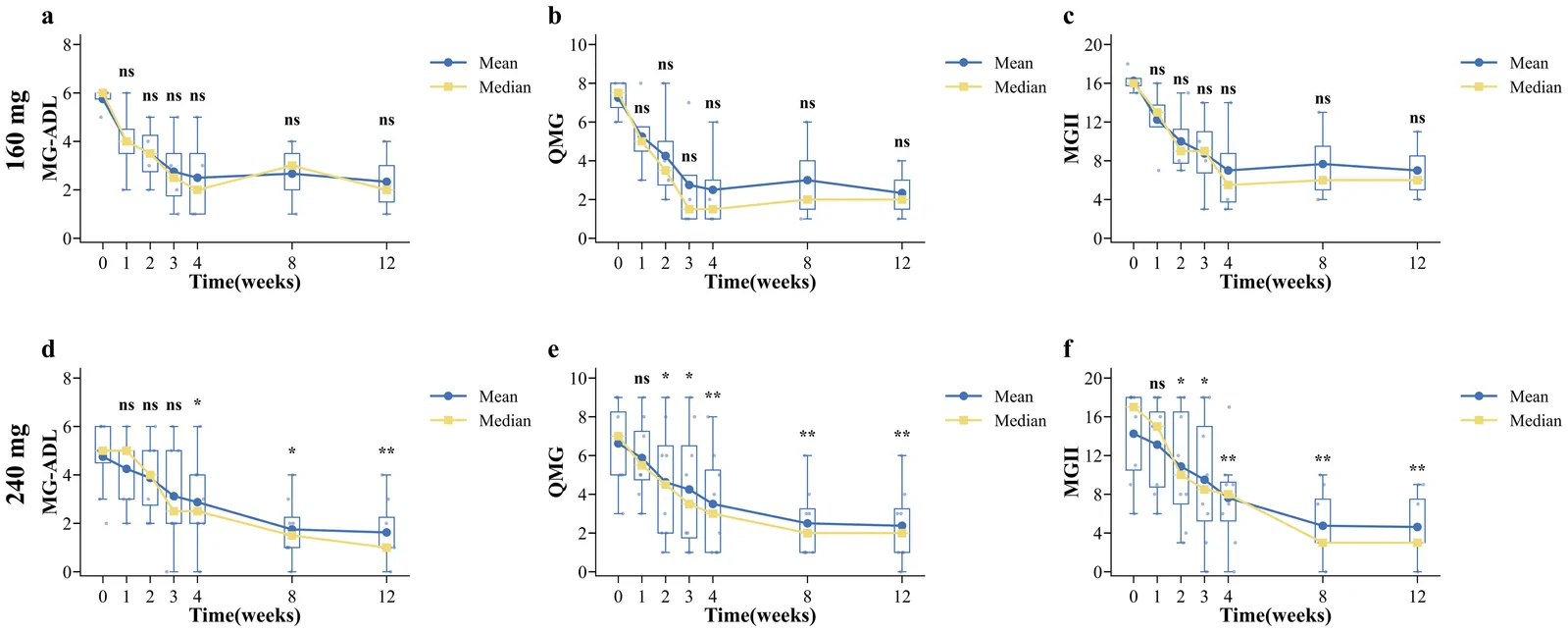
Comparison of clinical outcomes between 160 mg and 240 mg telitacicept dosing regimens in refractory ocular myasthenia gravis. **(a–c)** Changes from baseline to week 12 in Myasthenia Gravis Activities of Daily Living (MG-ADL), Quantitative Myasthenia Gravis (QMG), and Myasthenia Gravis Impairment Index (MGII) scores in patients administered telitacicept 160 mg weekly. **(d–f)** Changes from baseline to week 12 in MG-ADL, QMG, and MGII scores in patients treated with telitacicept 240 mg weekly. *P<0.05, **P<0.01.

### Comparison between telitacicept 160 mg and 240 mg

Among the 12 patients included in the analysis, 4 received telitacicept at 160 mg and 8 received 240 mg weekly at treatment initiation, respectively. By week 4, MSE was achieved by 2 cases in the 160 mg group versus 1 in the 240 mg group. From baseline to week 12, MSE was detected in 2 of 4 patients (50.0%) administered 160 mg and in 5 of 8 patients (62.5%) treated with 240 mg (**Figure 1**). Although the 160 mg group did not exhibit statistical significance, the improvement trends were evident for all disease scores. Conversely, the 240 mg group demonstrated significant reductions in QMG and MGII scores by week 2 (QMG: median reduction = 2.0, 95% CI 0.5–3.0, *p* = 0.031, r = 0.79; MGII: median reduction = 3.0, 95% CI 1.0–6.0, *p* = 0.031, r = 0.78), with MG-ADL scores showing a parallel downward trend at that time point (median reduction = 1.0, 95% CI 0.0–1.5, *p* = 0.063, r = 0.73). These score reductions in the 240 mg group were sustained through week 12 across all applied scales (all *p* < 0.05; all r > 0.85; **Figure 4**). Given the small sample sizes, all subsequent statistical comparisons between the two dose groups only illustrated symptomatic improvement trends without sufficient statistical power to confirm intergroup differences.

### Laboratory assessments

Longitudinal immunological analyses were performed in 11 of the 12 patients at baseline and at weeks 4, 8, and 12 following telitacicept initiation. One patient was excluded from biomarker analyses for having only a single baseline sample. Notably, two patients had recently received efgartigimod prior to enrollment, which likely contributed to their low baseline IgG levels: Patient 2 was administered their last dose 10 days before telitacicept (IgG 3.7 g/L), while Patient 12 received theirs 61 days prior (IgG 5.64 g/L).

**Figure 5:**
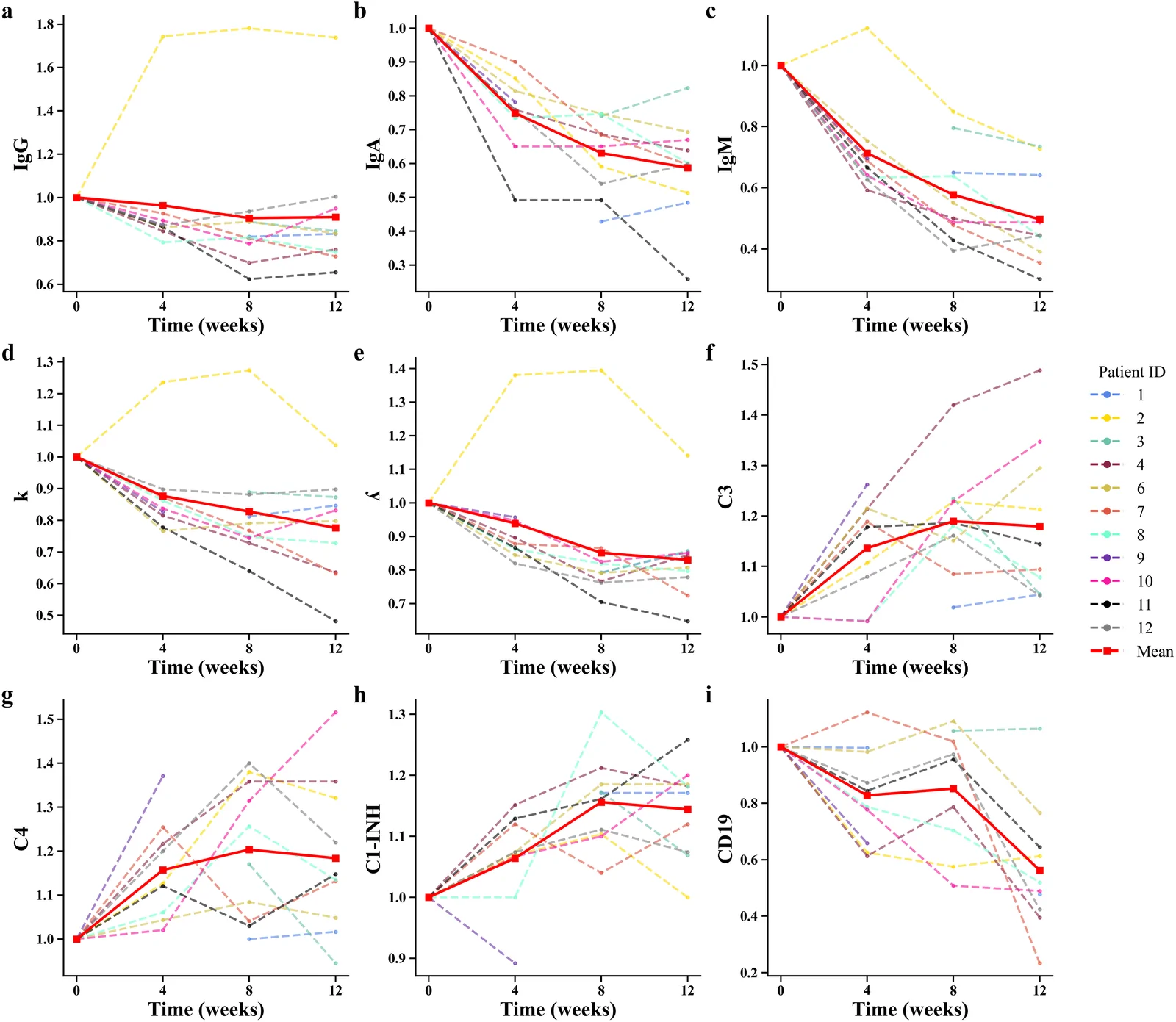
Longitudinal changes in immunological biomarkers during telitacicept treatment. **(a–c)** Mean and individual percentage changes in serum immunoglobulin G (IgG), immunoglobulin A (IgA), and immunoglobulin M (IgM) levels over the study period. **(d, e)** Mean and individual percentage changes in κ and λ immunoglobulin light chain levels, respectively. **(f–h)** Mean and individual percentage changes in the complement components C3c, C4, and C1 inhibitor over the study period. **(i)** Mean and individual percentage changes in circulating CD19^+^ B-cells over the study period.

Throughout the study period, most patients demonstrated a declining trend in serum immunoglobulin levels, including IgG, IgA, and IgM. At week 4, the median reductions from baseline were 12.9% for IgG, 24.1% for IgA, and 33.3% for IgM. By week 12, immunoglobulin levels remained below baseline, with median reductions of 16.7% for IgG, 40.1% for IgA, and 55.6% for IgM (**Figure 5a-c**).

Analysis of immunoglobulin light chains revealed similar patterns, with κ and λ light chain levels decreasing over the course of treatment (**Figures 5d, e**). In parallel, serum levels of complement components exhibited a consistent upward trend. By week 12, complement component C3c, complement component C4, and C1 inhibitor levels increased by approximately 17.9%, 18.4%, and 14.4%, respectively, relative to baseline (**Figures 5f–h**).

The proportion of circulating CD19^+^ B-cells decreased by 17.2% from baseline to week 4. Although a relative rebound to 14.8% reduction at week 8, the overall trajectory of CD19^+^ B-cell proportion over the 12-week treatment period was characterized by a net decline (**Figure 5i**).

### Safety

Telitacicept was generally well tolerated throughout the treatment period. Mild injection-site reactions, including localized swelling and pain, were reported in 6 of 12 patients (50.0%); these events were transient and resolved spontaneously within 1–2 days without medical intervention. No cases of anaphylaxis, clinically significant infections, or serious AEs were recorded. Importantly, no patient discontinued telitacicept treatment because of TRAEs during the study period (**Supplementary Table 3**).

## Discussion

In this retrospective case series, telitacicept was associated with rapid and sustained clinical improvement in patients with refractory OMG, a patient population for whom no biologic therapies are currently approved (9). Treatment with telitacicept was associated with consistent and clinically meaningful reductions in ocular symptom severity, as reflected by improvements in MGII, MG-ADL and QMG scores. Notably, symptomatic improvement was detected as early as the first week of treatment and was accompanied by substantial corticosteroid- and immunosuppressant-sparing effects achieved by week 8. In addition, telitacicept was well tolerated, with no serious AEs or treatment discontinuations, and only mild, self-limiting injection-site reactions reported. Collectively, these preliminary findings suggest that telitacicept may represent an effective and safe therapeutic option for patients with refractory OMG and provide a rationale for further prospective investigation in this patient population.

A notable finding of the present study is the rapid onset of clinical benefits following telitacicept initiation, with statistically significant reductions in ocular symptoms detected as early as one week after treatment initiation. This contrasts prior studies of GMG, in which clinically meaningful responses to telitacicept typically emerged after 4 weeks of therapy (17). Several factors may account for this apparent discrepancy. First, the intensive assessment schedule employed in this study, particularly during the early treatment phase, may have enabled early detection of therapeutic effects that were not captured in previous trials. Second, extraocular muscles are uniquely susceptible to neuromuscular transmission failure and may therefore exhibit more immediate functional recovery in response to immunomodulatory intervention. These considerations suggest that the ocular manifestations of MG may be especially sensitive to upstream B-cell-targeted therapies such as telitacicept. Importantly, improvements in ocular symptom severity were sustained and, in many patients, continued to deepen over the 12-week follow-up period, accompanied by progressive reductions in corticosteroid and immunosuppressant requirements. This pattern of sustained improvement is consistent with the proposed mechanism of telitacicept, which targets B-cell survival and plasma cell differentiation rather than transiently neutralizing circulating antibodies (16). Taken together, these observations support the potential role of telitacicept not only as a rapidly acting agent but also as a feasible long-term maintenance therapy for patients with refractory OMG, warranting further confirmation in prospective studies.

For patients with refractory OMG, the formulation of personalized treatment regimens is particularly crucial, and we have conducted preliminary explorations in this regard. The symptomatic phenotype of ocular MG typically consists of ptosis, diplopia, or their co-occurrence. Patients exhibited a notable trend toward improvement in ptosis as early as week 1, whereas meaningful improvement in diplopia was generally observed later. This differential response may be attributed to the distinct pathophysiological complexity involved. Ptosis primarily involves the dysfunction of the levator palpebrae superioris muscle. In contrast, the coordination of clear vision requires the precise and synchronized action of multiple extraocular muscles innervated by different cranial nerves (oculomotor, trochlear, and abducens). The intricate neural coordination required to maintain ocular alignment is more susceptible to fatigable weakness characteristic of MG and may therefore respond more slowly to immunomodulatory therapy. Nonetheless, by week 4 of telitacicept treatment, both symptoms showed marked and highly significant improvements. In our descriptive dose comparisons, patients administered telitacicept at 240 mg showed a trend toward more rapid onset of clinical improvement than those treated with 160 mg, a pattern consistent with observations from the phase II GMG trial in which both doses demonstrated efficacy (17). Through this exploratory subgroup analysis of ocular symptoms and dose response, we aimed to assess the potential use of telitacicept in personalized treatment strategies for MG. Such an approach may theoretically reduce the time to effective therapy and alleviate the associated economic burden for patients. However, the small sample size of the current study substantially limited its statistical power, precluding definitive conclusions regarding the dose-response relationship and its specific impact on ocular symptom improvement. Notably, the availability of a lower-cost 160 mg dose may improve treatment accessibility in resource-limited settings and warrants further investigation in prospective, dose-comparison trials.

In this study, telitacicept was associated with clinical improvements in cases with previous failure of other biologic therapies, including FcRn antagonists and complement inhibitors, underscoring the biological heterogeneity of MG pathogenesis. The pathogenic effects of AChR antibodies are mediated through multiple, non-mutually exclusive mechanisms, including complement-dependent postsynaptic damage, antibody-induced receptor internalization, and direct functional blockade of the AChR (19, 20). These mechanisms are heterogeneously distributed across patients, reflecting differences in antibody specificity, immunoglobulin subclasses, and downstream immune activation pathways. Consequently, therapies that selectively target a single pathogenic axis, such as complement inhibition or IgG recycling blockade, may be insufficient in a subset of patients, a limitation reflected in real-world data showing inadequate responses in 20-49.1% of treated individuals (21). In the present cohort, several patients who showed suboptimal or unsustained responses to FcRn inhibitors and/or complement C5 inhibitors subsequently achieved meaningful clinical improvement following telitacicept therapy. These individual treatment trajectories support the concept that antibody-driven, complement-independent mechanisms may predominate in certain refractory cases. Epidemiological and mechanistic studies further indicate that while complement-mediated injury accounts for a substantial proportion of MG pathology, AChR antibody-mediated receptor internalization and functional blockade are also highly prevalent, affecting up to 63-90% of patients (22, 23). In this context, telitacicept-dependent upstream targeting of B-cell survival and plasma cell differentiation, through simultaneous inhibition of BAFF and APRIL, may provide broader immunomodulatory coverage across diverse pathogenic pathways. By suppressing the production of multiple immunoglobulin isotypes and antibody subclasses rather than neutralizing a single effector mechanism, telitacicept may offer a therapeutic advantage for patients with refractory disease characterized by complex or overlapping immune drivers.

Treatment with telitacicept was associated with a modest overall reduction in circulating CD19^+^ B-cell, accompanied by dynamic changes in humoral immune markers. Prior studies have shown that B cells contribute to MG pathogenesis not only through autoantibody production but also via cytokine secretion and antigen presentation, which may explain why CD20-directed B-cell depletion with rituximab does not consistently result in parallel reductions in autoantibody titers (24). In contrast, telitacicept was associated with decreases in serum IgG, IgA, and IgM levels, suggesting broader suppression of antibody-producing pathways. IgG remains the principal pathogenic immunoglobulin in MG, whereas IgM is closely linked to complement activation; notably, approximately 12% of patients harbor coexisting AChR-specific IgG and IgM antibodies (19). In addition, immunoglobulin light chain analysis demonstrated alterations in κ free light chain levels, which were reported to be preferentially associated with OMG (25). Interestingly, treatment with telitacicept was also accompanied by increases in circulating complement components, including C3, C4, and C1 inhibitor, a finding that may reflect normalization of complement consumption rather than enhanced activation. Excessive complement activation is a core pathogenic mechanism in AChR antibody-positive MG, leading to immune complex deposition and structural damage at the neuromuscular junction. The detected complement changes therefore raise the hypothesis that telitacicept may indirectly modulate complement homeostasis by reducing upstream autoantibody production. Similarly, the insufficient clinical response observed in some patients treated with FcRn inhibitors may plausibly relate to their inability to reduce IgM levels or directly affect complement activity; however, this interpretation remains speculative and requires dedicated mechanistic studies. Collectively, these exploratory biomarker findings support the concept that telitacicept may exert a more comprehensive immunomodulatory effect by targeting multiple B-cell-dependent pathways and immunoglobulin isotypes, although further experimental and longitudinal studies are warranted to determine the precise biological mechanisms underlying these observations.

From a safety perspective, telitacicept appeared to be well tolerated in this cohort of patients with refractory OMG. No serious AEs, including severe infections, were recorded, and no AE-related treatment discontinuations occurred. The most frequently reported AEs were mild, transient injection-site reactions, which resolved spontaneously without intervention and did not interfere with the treatment courses. These observations are consistent with the safety profile reported in clinical trials of telitacicept in GMG and other autoimmune conditions (17, 26–28), supporting its suitability for outpatient administration and potential long-term use. No clinically meaningful immunosuppression-related complications were detected despite reductions in serum immunoglobulin levels, although longer follow-up is required to fully assess cumulative infection risk.

This study had several limitations that warrant consideration. First, as a retrospective, single-center, single-arm study, its design introduces selection bias and limits generalizability; additionally, the lack of a control group precludes definitive conclusion that the observed improvements are solely attributable to telitacicept. Consequently, all reported findings should be interpreted as hypothesis-generating rather than definitive causal evidence. Second, the small sample size restricts statistical power, particularly for exploratory subgroup and dose-comparison analyses, and precludes robust adjustment for potential confounders. Third, the relatively short follow-up duration does not allow assessment of long-term efficacy, durability of response, or delayed safety signals. In addition, initial dose selection of telitacicept was individualized based on clinical severity and socioeconomic considerations rather than standardized criteria, introducing treatment heterogeneity and limiting causal inference regarding dose-response relationships. Finally, although biomarker analyses were performed, their exploratory nature and incomplete longitudinal sampling constrain mechanistic interpretation.

In conclusion, this exploratory study suggests that telitacicept may represent a potentially effective and generally well-tolerated therapeutic option for patients with refractory OMG. Treatment was associated with a rapid onset of action, sustained improvement in ocular symptoms, and clinically meaningful steroid- and immunosuppressant-sparing effects, including in patients with previous failure of other biologic therapies. These preliminary findings support the potential role of telitacicept as an add-on therapy for refractory OMG. Prospective, randomized controlled, multicenter studies with larger sample sizes, longer follow-up, and systematic biomarker assessment are warranted to confirm these findings and to define optimal dosing strategies, maintenance regimens, and predictors of treatment response.

## Data Availability

The original contributions presented in the study are included in the article/**Supplementary Material**. Further inquiries can be directed to the corresponding author.

## References

[1] Marcus R . What is myasthenia gravis? JAMA. (2024) 331:452. doi: 10.1001/jama.2023.16872 38153702

[2] Garcia Estevez DA Pardo Fernandez J . Myasthenia gravis. Update on diagnosis and therapy. Med Clin (Barc). (2023) 161:119–27. doi: 10.1016/j.medcli.2023.04.006 37248131

[3] Li H Ruan Z Gao F Zhou H Guo R Sun C . Thymectomy and risk of generalization in patients with ocular myasthenia gravis: A multicenter retrospective cohort study. Neurotherapeutics. (2021) 18:2449–57. doi: 10.1007/s13311-021-01129-z 34625864 PMC8804035

[4] Shuey NH . Ocular myasthenia gravis: a review and practical guide for clinicians. Clin Exp Optom. (2022) 105:205–13. doi: 10.1080/08164622.2022.2029683 35157811

[5] Melson AT McClelland CM Lee MS . Ocular myasthenia gravis: updates on an elusive target. Curr Opin Neurol. (2020) 33:55–61. doi: 10.1097/wco.0000000000000775 31789705

[6] Kessi M Tang Y Chen B Wang G Zhang C He F . Pediatric ocular myasthenia gravis: Single-center experience. Pediatr Neurol. (2024) 153:137–43. doi: 10.1016/j.pediatrneurol.2024.01.014 38382246

[7] Guo RJ Gao T Ruan Z Zhou HY Gao F Xu Q . Risk factors for generalization in patients with ocular myasthenia gravis: A multicenter retrospective cohort study. Neurol Ther. (2022) 11:73–86. doi: 10.1007/s40120-021-00292-x 34729706 PMC8857387

[8] Ruan Z Guo R Zhou H Gao F Lin Y Xu Q . Association of immunosuppression treatment with generalization among patients with ocular myasthenia gravis: a propensity score analysis. Eur J Neurol. (2022) 29(6):1805–14. doi: 10.1111/ene.15292 35188698

[9] Narita T Nakane S Nagaishi A Minami N Niino M Kawaguchi N . Immunotherapy for ocular myasthenia gravis: an observational study in Japan. Ther Adv Neurol Disord. (2023) 16:17562864231163819. doi: 10.1177/17562864231163819 37051222 PMC10084546

[10] Blair HA . Efgartigimod: a review in generalised myasthenia gravis. Drugs. (2024) 84:1463–74. doi: 10.1007/s40265-024-02101-9 39511131

[11] Dhillon S . Eculizumab: a review in generalized myasthenia gravis. Drugs. (2018) 78:367–76. doi: 10.1007/s40265-018-0875-9 29435915 PMC5845078

[12] Yi JS Guptill JT Stathopoulos P Nowak RJ O'Connor KC . B cells in the pathophysiology of myasthenia gravis. Muscle Nerve. (2018) 57:172–84. doi: 10.1002/mus.25973 28940642 PMC5767142

[13] Abeles I Palma C Meednu N Payne AS Looney RJ Anolik JH . B cell-directed therapy in autoimmunity. Annu Rev Immunol. (2024) 42:103–26. doi: 10.1146/annurev-immunol-083122-044829 38011889

[14] Dos Santos A Noury JB Genestet S Nadaj-Pakleza A Cassereau J Baron C . Efficacy and safety of rituximab in myasthenia gravis: a French multicentre real-life study. Eur J Neurol. (2020) 27(11):2277–85. doi: 10.1111/ene.14391 32526053

[15] Haghikia A Hegelmaier T Wolleschak D Bottcher M Desel C Borie D . Anti-CD19 CAR T cells for refractory myasthenia gravis. Lancet Neurol. (2023) 22:1104–5. doi: 10.1016/s1474-4422(23)00375-7 37977704

[16] Dhillon S . Telitacicept: first approval. Drugs. (2021) 81:1671–5. doi: 10.1007/s40265-021-01591-1 34463932

[17] Yin J Zhao M Xu X Zhang M Xu Z Li Z . A multicenter, randomized, open-label, phase 2 clinical study of telitacicept in adult patients with generalized myasthenia gravis. Eur J Neurol. (2024) 31:e16322. doi: 10.1111/ene.16322 38726639 PMC11235933

[18] Lin J Li Y Gui M Bu B Li Z . Add-on telitacicept significantly improves outcome of patients with refractory ocular myasthenia gravis a real-world case series. Brain Behav. (2026) 16:e71157. doi: 10.1002/brb3.71157 41476025 PMC12755965

[19] Khani-Habibabadi F Roy B Pham MC Obaid AH Filipek B Nowak RJ . AChR autoantibody pathogenic properties are heterogeneously distributed and undergo temporal changes among patients with myasthenia gravis. Neurol Neuroimmunol Neuroinflamm. (2025) 12:e200436. doi: 10.1212/nxi.0000000000200436 40680247 PMC12275905

[20] Bayer AC Sanmarco LM Pellerin A Masi G Plasencia A Anderson JM . Therapeutic IgG- and IgM-specific proteases disarm the acetylcholine receptor autoantibodies that drive myasthenia gravis pathology. Proc Natl Acad Sci USA. (2025) 122:e2505984122. doi: 10.1073/pnas.2505984122 41118207 PMC12582293

[21] Huntemann N Gerischer L Herdick M Nelke C Stascheit F Hoffmann S . C5 complement inhibition versus FcRn modulation in generalised myasthenia gravis. J Neurol Neurosurg Psychiatry. (2025) 96:310–21. doi: 10.1136/jnnp-2024-334404 39798960 PMC12015038

[22] Obaid AH Zografou C Vadysirisack DD Munro-Sheldon B Fichtner ML Roy B . Heterogeneity of acetylcholine receptor autoantibody-mediated complement activity in patients with myasthenia gravis. Neurol Neuroimmunol Neuroinflamm. (2022) 9(4):e1169. doi: 10.1212/NXI.0000000000001169 35473886 PMC9128035

[23] Drachman DB Adams RN Josifek LF Self SG . Functional activities of autoantibodies to acetylcholine receptors and the clinical severity of myasthenia gravis. N Engl J Med. (1982) 307:769–73. doi: 10.1056/nejm198209233071301 7110241

[24] Du Y Li C Hao YF Zhao C Yan Q Yao D . Individualized regimen of low-dose rituximab monotherapy for new-onset AChR-positive generalized myasthenia gravis. J Neurol. (2022) 269:4229–40. doi: 10.1007/s00415-022-11048-4 35243555

[25] Wilf-Yarkoni A Alkalay Y Brenner T Karni A . High kappa free light chain is a potential biomarker for double seronegative and ocular myasthenia gravis. Neurol Neuroimmunol Neuroinflamm. (2020) 7(5):e831. doi: 10.1212/nxi.0000000000000831 32665296 PMC7371374

[26] van Vollenhoven RF Wang L Merrill JT Liu Y Bao C Li F . A phase 3 trial of telitacicept for systemic lupus erythematosus. N Engl J Med. (2025) 393:1475–85. doi: 10.1056/nejmoa2414719 41092329

[27] Xu D Fang J Zhang S Huang C Huang C Qin L . Efficacy and safety of telitacicept in primary Sjogren's syndrome: a randomized, double-blind, placebo-controlled, phase 2 trial. Rheumatol (Oxford). (2024) 63:698–705. doi: 10.1093/rheumatology/kead265 37399108

[28] Wu D Li J Xu D Merrill JT van Vollenhoven RF Liu Y . Telitacicept in patients with active systemic lupus erythematosus: results of a phase 2b, randomised, double-blind, placebo-controlled trial. Ann Rheum Dis. (2024) 83:475–87. doi: 10.1136/ard-2023-224854 38129117 PMC10958275

