# Supplementary material for: Effectiveness and safety of telitacicept in refractory ocular myasthenia gravis: a retrospective, real-world study
Source: Front Immunol. 2026 Sep 3;17:1797461. doi: 10.3389/fimmu.2026.1797461 (PMC13582499; doi:10.3389/fimmu.2026.1797461)
Supplement: Supplementary file 1 [file Table1.docx]

**Supplementary figures and tables**

**Supplementary Table 1.** Immunotherapeutic history and treatment rationale in patients with refractory ocular myasthenia gravis

| Patient | Prior immunotherapies | Immunotherapy at telitacicept initiation | Reasons for telitacicept initiation |
| --- | --- | --- | --- |
| 1 | Corticosteroids, tacrolimus, mycophenolate mofetil | Corticosteroids, tacrolimus | Inadequate response to standard immunotherapies; marked impairment in quality of life |
| 2 | Corticosteroids, tacrolimus, efgartigimod, rituximab | Corticosteroids, tacrolimus | Refractory to both standard and biologic therapies; marked impairment in quality of life |
| 3 | Corticosteroids, tacrolimus | Tacrolimus | Corticosteroid-related adverse effects; inadequate response to standard therapies; marked impairment in quality of life |
| 4 | Corticosteroids, tacrolimus | Corticosteroids | Corticosteroid-related adverse effects; refractory to standard therapies; marked impairment in quality of life |
| 5 | Corticosteroids, tacrolimus, efgartigimod | Corticosteroids, tacrolimus | Refractory to standard and biologic therapies; marked impairment in quality of life |
| 6 | Corticosteroids, tacrolimus | Tacrolimus | Corticosteroid-related adverse effects; inadequate response to standard therapies; marked impairment in quality of life |
| 7 | Corticosteroids, tacrolimus, efgartigimod, eculizumab | Corticosteroids | Refractory to standard and biologic therapies; efgartigimod-associated hypogammaglobulinemia; marked impairment in quality of life |
| 8 | Corticosteroids, tacrolimus, azathioprine, eculizumab | Corticosteroids, tacrolimus | Refractory to standard and biologic therapies; marked impairment in quality of life |
| 9 | Corticosteroids, tacrolimus, azathioprine | Corticosteroids, tacrolimus | Tacrolimus-related adverse effects; marked impairment in quality of life |
| 10 | Corticosteroids, tacrolimus | Corticosteroids, tacrolimus | Corticosteroid-related adverse effects; inadequate response to standard immunotherapies; marked impairment in quality of life |
| 11 | Corticosteroids, tacrolimus, azathioprine | Corticosteroids, tacrolimus | Inadequate response to standard immunotherapies; marked impairment in quality of life |
| 12 | Corticosteroids, tacrolimus, efgartigimod | Corticosteroids, tacrolimus | Refractory to standard and biologic therapies; marked impairment in quality of life |

**Supplementary Table 2.** Proportion of missing outcome data at each follow-up time point

| Outcome | Baseline | Week1 | Week2 | Week3 | Week4 | Week8 | Week12 |
| --- | --- | --- | --- | --- | --- | --- | --- |
| ADL | 0（0） | 0（0） | 0（0） | 0（0） | 0（0） | 1（8.3%） | 1（8.3%） |
| QMG | 0（0） | 0（0） | 0（0） | 0（0） | 0（0） | 1（8.3%） | 1（8.3%） |
| MGII | 0（0） | 0（0） | 0（0） | 0（0） | 0（0） | 1（8.3%） | 1（8.3%） |
| prednisone daily dose | 0（0） |  |  |  | 0（0） | 1（11.1%） | 1（11.1%） |
| tacrolimus daily dose | 0（0） |  |  |  | 0（0） | 1（10%） | 1（10%） |
| IgG | 1（8.3%） |  |  |  | 3（25%） | 1（8.3%） | 2（16.7%） |
| IgA | 1（8.3%） |  |  |  | 3（25%） | 1（8.3%） | 2（16.7%） |
| IgM | 1（8.3%） |  |  |  | 3（25%） | 1（8.3%） | 2（16.7%） |
| κ light chain | 1（8.3%） |  |  |  | 3（25%） | 1（8.3%） | 2（16.7%） |
| λ light chain | 1（8.3%） |  |  |  | 3（25%） | 1（8.3%） | 2（16.7%） |
| C3c | 1（8.3%） |  |  |  | 3（25%） | 1（8.3%） | 2（16.7%） |
| C4 | 1（8.3%） |  |  |  | 3（25%） | 1（8.3%） | 2（16.7%） |
| C1-INH | 1（8.3%） |  |  |  | 3（25%） | 1（8.3%） | 2（16.7%） |
| CD19+B | 1（8.3%） |  |  |  | 2（16.7%） | 3（25%） | 2（16.7%） |

**Supplementary Table 3.** Summary of Adverse Events in All Patients (n=12)

| Patient | Upper respiratory tract infection | Anaphylaxis | Injection site reactions | Number of injections | Serious adverse event | Adverse event leading to discontinuation |
| --- | --- | --- | --- | --- | --- | --- |
| 1 | 0 | 0 | 0 | 12 | 0 | 0 |
| 2 | 0 | 0 | 4 | 12 | 0 | 0 |
| 3 | 0 | 0 | 0 | 12 | 0 | 0 |
| 4 | 0 | 0 | 0 | 12 | 0 | 0 |
| 5 | 0 | 0 | 3 | 12 | 0 | 0 |
| 6 | 0 | 0 | 2 | 12 | 0 | 0 |
| 7 | 0 | 0 | 2 | 12 | 0 | 0 |
| 8 | 0 | 0 | 0 | 12 | 0 | 0 |
| 9 | 0 | 0 | 0 | 4 | 0 | 0 |
| 10 | 0 | 0 | 1 | 12 | 0 | 0 |
| 11 | 0 | 0 | 1 | 12 | 0 | 0 |
| 12 | 0 | 0 | 0 | 12 | 0 | 0 |

**Supplementary Fig. 1.** Changes in tacrolimus dosage during telitacicept treatment. (A) Mean and (B) individual daily tacrolimus doses from baseline through week 12. *P<0.05.
